# A Randomised-Controlled Study Demonstrates That Diet Can Contribute to the Clinical Management of Feline Atopic Skin Syndrome (FASS)

**DOI:** 10.3390/ani15101429

**Published:** 2025-05-15

**Authors:** Adrian Watson, Jeremy Laxalde, Thomas Brément, Emilie Vidémont Drevon-Gaillot, Marion Mosca, Elisa Maina, Xavier Langon

**Affiliations:** 1 Royal Canin SAS, 30740 Aimargues, France; jeremy.laxalde@royalcanin.com (J.L.); xavier.langon@effem.com (X.L.); 2 Veterinary Dermatology Referral Service EIRL Vet’Dermathome, 83190 Ollioules, France; vetdermathome@yahoo.com; 3 Centre Hospitalier Vétérinaire Saint-Martin, 321 Impasse des Champs, 74350 Allonzier la Caille, France; emiliedrevon@gmail.com; 4 VetAgro Sup, Interactions Cells Environment, UPSP 2016.A104, Université de Lyon, 69280 Marcy L’étoile, France; marion.mosca@vetagro-sup.fr; 5 Department of Clinical Veterinary Medicine, Clinical Dermatology, Vetsuisse Faculty, University of Bern, CH-3012 Bern, Switzerland; elisa.maina@unibe.ch

**Keywords:** cat, allergy, nutrition, dermatitis, pruritus, inflammation

## Abstract

Feline atopic skin syndrome (FASS) is a common inflammatory and highly pruritic skin condition. It typically manifests according to one or more of four different clinical lesion patterns: miliary dermatitis, self-induced alopecia, head and neck pruritus, and eosinophilic granuloma complex. FASS is a chronic and relapsing condition that often persists for life. Effective management can be a significant challenge for both clinicians and owners. Here, we investigated whether diet could act as an adjunct to conventional medicated therapies to improve the management of FASS. We conducted a six-month randomised, placebo-controlled, double-blinded study on client-owned FASS cats to test a novel pet food formulation. The main variables followed were the veterinarian (FeDESI) and the owner (VAScat) assessing symptom severity, in addition to medication requirements. Compared to the control diet, the test diet not only ameliorated symptoms but also significantly reduced the dependency on medication. The VAScat metric was improved after 3 months (*p* = 1.6 × 10^−4^) and 6 months (*p* = 5.7 × 10^−5^) in the test group only, and the cats’ medication requirement significantly declined exclusively in the test group from month 2 onwards. The interpretation of the FeDESI results was hampered by high levels of variation, particularly in the control group. Nonetheless, in the test group, clinical scores showed evidence of improvement after 3 months (*p* = 0.037) and 6 months (*p* = 0.02), and no such improvement was detected in the control group. Overall, our findings suggest that tailored nutrition can significantly aid in the management of feline atopic skin syndrome.

## Simple Summary

Feline atopic skin syndrome (FASS) is a common inflammatory and highly pruritic skin condition. We evaluated the benefits of a novel diet, working alongside conventional treatments, fed for six months to cats with FASS. We collected data on the veterinarian and owner-assessed symptom severity, as well as information about the medication required by the cats to control the condition. We discovered that the diet was able to reduce the medication burden of the cats over six months, as well as showing evidence of reducing symptoms. We conclude that diet can be valuable in the clinical management of FASS.

## 1. Introduction

Feline Atopic Skin Syndrome (FASS) is an inflammatory and pruritic dermatitis in cats [1,2]. FASS is now the terminology used for what has also been described as feline atopy or “non-flea non-food induced feline hypersensitivity dermatitis” [2,3]. Affected cats present with one or more dermatological signs, all of which may or may not be associated with IgE antibodies against environmental allergens. These clinical reaction patterns include miliary dermatitis (MD), self-inflicted alopecia/hypotrichosis (SIAH), head and neck pruritus (HNP), and eosinophilic granuloma complex (EGC) [2,3,4], and occasionally, they are limited to diffuse pruritus alone. The clinical presentation of FASS differs significantly from that of canine atopic dermatitis (cAD), which has a more restricted range and distribution of lesions [4]. As observed in cAD, FASS seasonality can be observed, which contrasts with food allergy owing to seasonal variations in the levels of common allergens such as pollen and mould. Although little is known about the heritable component of FASS, a genetic contribution has been suggested by the apparent over-representation of certain breeds [2,5,6,7]. Patho-mechanistically, there are similarities between FASS and atopic conditions in other species, such as the overreaction of the immune system to environmental allergens. Immunoglobulin E may also play a role in disease development, characterised by inflammation and accompanied by activation of eosinophils and lymphocytes. In at least a proportion of cats, the inflammation associated with FASS indicates T-helper type 2 immune dysregulation, akin to other forms of AD [8]. The skin barrier of cats with FASS may be more sensitive to allergens, with potential involvement of a genetic component [9]; however, evidence for this is more limited than that in other species.

Currently, no specific tests are available for the diagnosis or symptomatic monitoring of FASS. Diagnosis relies on a clinical process based on the presence of compatible signs and the exclusion of diseases sharing similar features [4]. Elimination or exclusion of fleas/flea allergy, other parasites, infections, and food allergies is mandatory. The severity assessment of FASS lesions associated with the four main presentations relies on clinical scoring methods. One of the most commonly used is the FeDESI (Feline Dermatitis Extent and Severity Index), which evaluates the severity of erythema, excoriations/erosions, and self-induced alopecia at 42 body sites using a 0–5 point scale (maximum score 630; [10,11]). Veterinarian-assessed FeDESI can be complemented by simpler measures such as the dual feline pruritus Visual Analogue Scale (VAScat) [12], which is a validated mechanism for capturing owner perception of pruritus based on cat behaviour. When assessing the efficacy of combination therapies for complex conditions in dogs, there has been an increasing application of the Medication Score in clinical studies. This method collates information regarding the type, dose, and duration of medications required to maintain an adequate quality of life for an animal. The data were then used as a metric to evaluate multimodal disease management [13]. Although the medication score has not previously been used in feline FASS studies, the same principle could reasonably be applied, given certain adjustments for this species.

The use of diet to complement more conventional approaches for managing atopic dermatoses, such as medication and immunotherapy, has previously shown some promising results. Much of what we now know about the use of nutrition to help human patients with atopic dermatitis has been comprehensively reviewed elsewhere [14]. Notable examples are vitamin A and vitamin D, which have shown benefits via immunoregulatory mechanisms, albeit at elevated doses. Omega-6 (skin barrier) and omega-3 fatty acids (anti-inflammatory mediators) have shown promise, and zinc has been demonstrated to confer a number of benefits. Antioxidants, such as vitamins E and C, appear to be beneficial, likely due to their ability to counter the pro-oxidative environment of skin lesions [15]. Two recent canine studies have indicated that conventional nutrients, such as those listed above, can be complemented by phytotherapeutic compounds. One study showed that a pet food containing antioxidants, omega-3 and -6 polyunsaturated fatty acids, and two phytochemical extracts was able to significantly reduce the medication required to maintain control over cAD symptoms over an extended period [16]. In another study, a diet containing a combination of antioxidants and polyunsaturated fatty acids, with the addition of polyphenol sources, showed promise as a supportive agent for the management of canine atopic dermatitis [17]. To date, there has been less interest in investigating the role of nutritional support in feline atopic conditions such as FASS. Some studies have shown promise but have not been able to provide strong evidence of efficacy [18,19]. One well-controlled experiment performed to investigate the potential of orally dosed ultramicronised palmitoylethanolamide (uPEA) to maintain remission in cats with non-flea hypersensitivity dermatitis showed positive results [20]. In addition to increasing the interval time between relapses, there was also a reduction in pruritus severity when the cats’ symptoms returned. A further study into a dry food containing supplemented ω3-fatty acids, γ-linolenic acid, and other nutrients showed evidence of improved coat quality and reduced pruritus in 11 non-flea, non-food-induced hypersensitivity dermatitis cats [21].

Here, we describe a randomised, placebo-controlled, double-blinded study to explore the potential of a therapeutic diet to augment drug-based strategies conventionally used to manage FASS. The therapeutic diet tested included augmented concentrations of a number of the antioxidant and anti-inflammatory components previously tested in humans and canines, as well as a phytochemical extract from turmeric root. In this instance, omega-3 Polyunsaturated Fatty Acids (PUFAs), docosahexaenoic acid (DHA), and eicosapentaenoic acid (EPA) were primarily delivered through marine algae oil.

## 2. Materials and Methods

### 2.1. Animals

The experimental cohort comprised client-owned cats aged 12 months or older, of any breed or sex, treated in dermatology specialist practices. Cats were diagnosed with FASS according to the guidelines published by Santoro et al. in 2021 [4], which is considered the most robust methodology at the time of writing. In brief, cats with a history of non-seasonal pruritus, after exclusion of ectoparasites and other diseases resembling such as bacterial and yeast infections, could be enrolled. Cats with active bacterial and/or yeast infections, as well as individuals responding adequately to an eight-week elimination diet (>50% improvement of symptoms), were not included. Cats undergoing allergen-specific immunotherapy for less than 12 months or cats controlled with this intervention alone were excluded. Cats with significant other pathologies, such as cancer, chronic kidney disease, hyperthyroidism, and autoimmune conditions, were also excluded. All cats required ongoing ectoparasite treatment throughout the study. Concomitant medications (see Table 1 for list), excluding all nutritional supplements, were permitted throughout the study to comply with ethical guidelines and to ensure an optimal quality of life. These treatments were recorded in a study diary.

### 2.2. Initial Clinical Scoring at Inclusion

All enrolment and clinical evaluations were performed by Board-certified specialists in veterinary dermatology. Cats were initially evaluated for skin lesions and pruritus using the Feline Dermatitis Extent and Severity Index (FeDESI scores) and a Visual Analogue Scale for feline pruritus severity scoring (VAScat), respectively. The medications used during the month prior to inclusion were recorded. The medication score was derived according to the system shown in Table 1. The medication score system was based on that of Fischer et al. [13], but with adaptations made for typical feline treatment regimes. Monthly treatments were recorded, with the drug, dosage level, and frequency of use attributed to a numerical score. The overall score for each month was determined by summing the daily scores and dividing them by the number of days in the month.

### 2.3. Follow-Up Consultations and Drop-Outs

Follow-up consultations were conducted according to the following schedule: a telephone consultation with the veterinarian one month after the start of the diet, followed by in-person consultations at three and six months, and additional telephone consultations at two, four, and five months. The purpose of the one-month consultation was to address any issues related to the study, such as food acceptance, and to record medication and pruritus scores. The in-person consultations at three and six months included a full examination of the cats, capturing FeDESI, VAScat, and Medication scores. Telephone consultations focused on capturing the VAScat and Medication scores. At all time points, the consumption of the foods was reported by the owners using a binary yes/no answer to establish if the cat was eating the diet well, with comments requested as needed. In addition, throughout the study, the owners were asked to evaluate the quality of the cats’ stools in order to assess diet tolerance. Stool quality was evaluated using a five-point scale, illustrated by an image (5 = hard to 1 = liquid diarrhoea; optimum 2.5).

Owners were permitted to withdraw their cats from the study at any time and for any reason. Drop-out reasons were recorded. Cats maintained on experimental diets for a minimum of three months were considered for data analysis.

### 2.4. Diets

The study was double-blinded and placebo-controlled in design. Cats were assigned to one of two study groups upon recruitment; one group received the test food, and the other received standard premium food. The two diets were given code names for blinding purposes and, aside from printed names, were packaged in identical neutral bags. The randomisation schedule was defined prior to the start of the study using an Excel spreadsheet. The schedule did not consider the symptomatic presentation of FASS cases (MD, SIAH, HNP, or EGC). Both diets were dry kibbles with similar size, colour, and general appearance, produced at a commercial manufacturing facility in France. The two diets were complete and balanced for adult cats at the maintenance energy requirement (95 kcal/kg^0.75^ NRC 2006) [22]. A comparison of the key nutritional components of the two diets is presented in Table 2. All ingredients were standard pet food materials, except for the turmeric extract, which was provided by Indena^®^ (Bengaluru, India) and contained 20% curcuminoids with enhanced bioavailability. The omega-6 linoleic acid content of the test diet was increased by replacing the majority of the pork fat in the control diet with soya oil and chicken fat. The omega-3 fatty acids were supplemented in the test diet primarily via marine algal oil, with a small portion (~5%) from fish oil. Beyond the feeding guidelines, neither the owners nor the investigators were provided with further details about the food. The owners were instructed to feed the animals the diet exclusively throughout the study. When transitioning from their previous diet to the study diets, owners were advised to change gradually over six days (2 days with 75%:25%; 2 days with 50%:50%; 2 days with 25%:75%).

### 2.5. Outcomes Measures

Drop-out reasons and unexpected events were recorded. In addition to the primary measures of FeDESI, VAScat, and Medication score, the percentages of cats with >50% improvement in the clinical score were computed for both groups. Additionally, the number of cats returning to normal/remission (considered FeDESI < 11) was recorded in both groups.

### 2.6. Statistical Methods

Powering for this study was performed using data from the closest comparable work investigating the role of diet in the clinical management of canine atopic dermatitis [16]. From a previous study, we used the means and standard deviations of medication score data after 6 months of diet for the test and control groups. Group size estimation was performed using an unpaired *t*-test for 80% power with an alpha risk of 0.003, corrected by the Bonferroni method for multiple comparisons. The estimated cat group size was 21.

Statistical analyses were performed using R Core Team, 2021 [23]. The dplyr package was used for data manipulation and ggplot [24] for data visualisation [25]. Linear mixed models were calculated using the lmer function from the lme4 [26] package, and the emmeans [27] package was used to calculate marginal means, pairwise contrasts, and effect sizes.

Linear mixed models were used to evaluate the impact of diet, time, and time by diet interaction on FeDESI, VAScat, and Medication score. The animal was modelled as a random term.

The statistical assumptions of the Linear Mixed Models were verified using the Shapiro-Wilk test for the normal distribution of residuals and graphically for the homoscedasticity of residuals. Pairwise comparisons were applied within time, between groups and within groups, and between time. Adjustments were made using Tukey’s HSD for multiple comparisons. The level of significance was set at 5%. The median (min-max) is provided for the studied parameters. The experimental unit was an individual cat. The analyses included all cats that received the study diet per protocol for any duration, except those lost to follow-up before month 2. Adverse events were reported in enrolled cats that received the study diet for any duration. Quoted Confidence Intervals (CIs) were modelled using the emmeans package.

## 3. Results

### 3.1. Study Population

A total of 27 cats (20 females and seven males; all sterilised) were recruited for the study by Board-certified specialists in veterinary dermatology (authors TB, EDG, EM, and MM) at veterinary clinics in France and Switzerland between August 2021 and August 2023. Written consent was obtained from the owners of all cats prior to their participation in the study. Of the cats recruited, two did not provide data beyond the first phone consultation and were not included in the data analysis; one cat from the control group and one from the test group. The control group cat was withdrawn due to a severe deterioration in symptoms. No reason was given for the withdrawal of the test group cat. Seventeen of the remaining 25 cats were described as European Short Hair, three as Maine Coons, two as Ragdolls, one as Abyssinian, one as Chartreux, and one as Siamese. The age at the time of recruitment ranged from 18 months to 14 years, with a median age of 4 years. The distribution of FASS clinical presentations at diagnosis was as follows: 10 HNP, 2 SIAH, 5 EGC, 5 SIAH/EGC, 2 HNP/SIAH, and 1 SIAH/MD. The distribution of the groups is shown in Table 3.

### 3.2. Data Collection from the Cohort

At inclusion, the mean FeDESI scores for the two groups were test, 19 (4–46), and control, 26 (6–60), with no significant difference between the groups (*p* = 0.23). The average VAScat at initiation was 7.3 (6–10) in the test group and 5.5 (3–8) in the control group, with no significant difference between the two (*p* = 0.085). The average wedication scores were 28 (5–70) for the test group and 27.5 (5–45) for the control group, with no significant difference between the two groups (*p* = 0.94). All twenty-five cats were available for FeDESI assessment at the 3-month timepoint, and 16 cats were available at six months (10 test vs. six control). Data for VAScat were collected from all 25 cats at 3 months and 21 cats at 6 months (13 test vs. eight control). Medication score data were not collected consistently at the 1-month timepoint but were more complete by 2 months. Therefore, the first medication score data reported were labelled as 1–2 months. At this time, there were 25 cats, and this number was maintained for the first 3 months. Subsequently, 23 cats were included at 4 months (15 test vs. eight control) and 21 cats at 5 months (13 test vs. eight control). Additional effort to contact owners at the end of the study resulted in the collection of medication score data from 23 cats (14 test vs. nine control). We determined that one female Ragdoll in the control group experienced a severe deterioration in disease severity, which led to the owner‘s withdrawal at 3 months. In another case, a European female in the test group was withdrawn at 4 months due to food refusal. Missing Medication score data points were due to the veterinarians’ inability to contact the owners.

The digestibility of the diets was assessed using a faecal scoring system. The results showed that both diets were well tolerated by the cats in both groups.

### 3.3. Evolution of Scores

#### 3.3.1. General Evolution

There was a significant effect of time on the FeDESI (*p* = 4.7 × 10^−3^), VAScat (*p* < 1 × 10^−4^), and medication scores (*p* < 1 × 10^−4^). There was a significant difference between the diet groups for FeDESI (*p* = 0.031), but no interaction between diet and time point for FeDESI (*p* = 0.6). Diet overall did not have a significant effect on VAScat and Medication score (*p* = 0.42 and *p* = 0.11, respectively), but there was a significant interaction between diet and time point for VAScat (*p* = 0.012) and medication score (*p* = 4 × 10^−3^).

**Figure 1:**
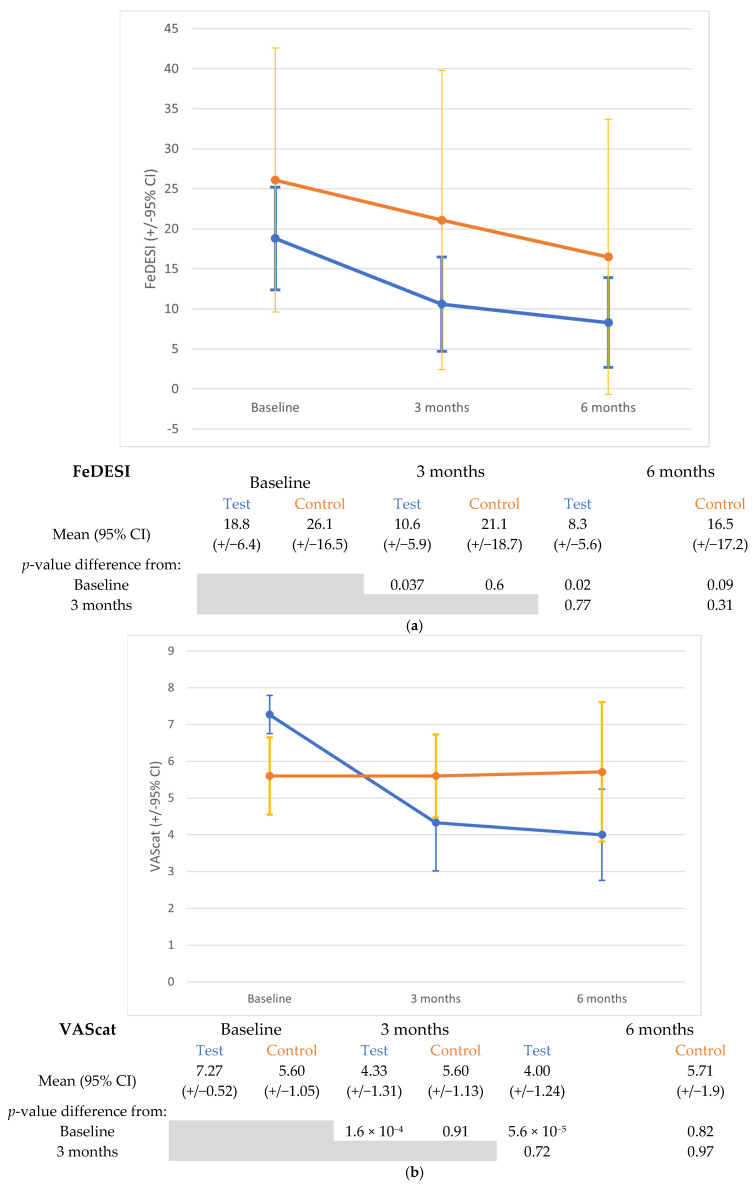

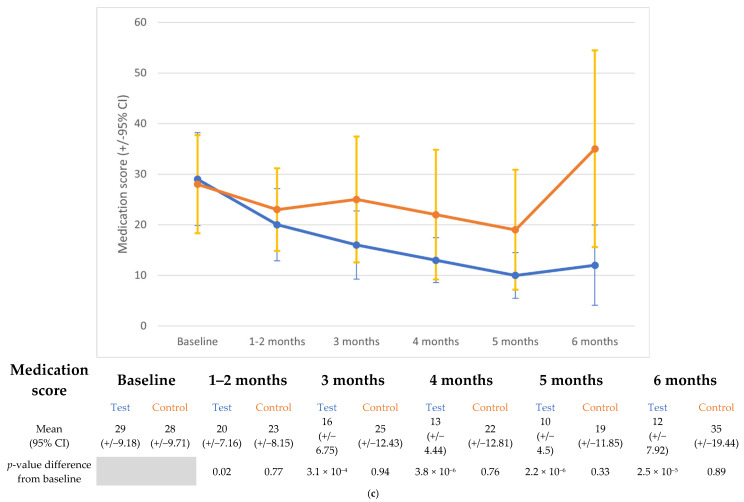
(**a**–**c**). FASS lesion severity, pruritus intensity, and medication requirements. Mean observed data (95% confidence interval) for (**a**) Feline Dermatitis Extent and Severity Index (FeDESI) score, (**b**) Visual Analogue Scale score (VAScat), and (**c**) Medication Score. Blue—Test diet; Orange—Control diet. The accompanying tables show the corresponding mean values (95% CI, calculated from data) and *p*-values for comparisons between 3 and 6 months vs. baseline and 3 vs. 6 months.

FeDESI, VAScat, and Medication scores were compared within each diet group between baseline and months 3 and 6 and between the two diet groups at each timepoint (Figure 1; Table 4 and Table 5).

#### 3.3.2. FeDESI

FeDESI decreased significantly between baseline and 3 months (*p* = 0.037) and 6 months (*p* = 0.02) in the test diet group, but not in the control group (Figure 1a; Table 4). At 3 months, the effect size was greater for the test group (1.01 (0.19–1.83)) than for the control group (0.46 (0.57–1.48] ), whereas at 6 months, the effect sizes were similar (test 1.33 (0.34–2.32) vs. control 1.42 (0.06–2.78)). When comparing FeDESI between test and control groups at each time point, there was no difference at baseline (*p* = 0.084), but FeDESI was significantly lower in the test group at 3 months (*p* = 0.013). No difference was detected at the 6-month timepoint (*p* = 0.2).

#### 3.3.3. VAScat

The VAScat owner pruritus assessment showed a significant decrease when comparing the baseline to both 3 months (*p* < 2 × 10^−4^) and 6 months (*p* < 1 × 10^−4^) within the test group, whereas no difference was observed in the control group (Figure 1b; Table 4). Effect sizes were higher in the test group at both 3 and 6 months, 1.71 (0.88–2.53) and 2.03 (1.1–3), respectively, versus the control group, 0.2 (−0.76–1.16) and 0.32 (−0.77–1.41). For VAScat, there was no difference between the two diet groups at baseline, 3, or 6 months (*p* = 0.11, *p* = 0.15, and *p* = 0.12, respectively).

### 3.4. Medication Score

Medication scores reveal a monthly assessment of disease status via the proxy of medication required for effective symptom management. When each monthly score was compared to the baseline, we observed a clear difference in the medication requirements for the two diet groups. There was a consistent, significant reduction in the average medication requirement relative to the baseline for the cats fed the test diet. This was observed as early as the time point of 2 months (*p* < 0.02) and was maintained for all subsequent time points until the end of the study (Figure 1c; Table 5). In contrast, for the control group, no change was observed in the average medication score at any time point relative to the baseline. Effect sizes were consistently higher for the test group than for the control group (range 1.15–2.14 versus range 0.41–1.02, respectively). When the two diet groups were compared at each timepoint, there was a significant difference between the two groups at month 6 (*p* = 2 × 10^−3^) only. At this time point, the test diet-fed cats were found to require significantly less medication than those in the control group.

A summary of the extent of clinical improvement in the two groups is presented in Table 6. In the test group, approximately half of the cats improved their FeDESI scores by at least 50% by the 3- and 6-month timepoints (7/15 and 5/10). In the control group, one-third of the cats (3/10) had improved by 50% or more at 3 months, which rose to 67% (4/6) by 6 months. A greater than 50% reduction in the owner-assessed VAScat score was seen in 40% (6/15) and 45% (5/11) of the test cats by 3 and 6 months, respectively. At the equivalent time points, the percentages were 10% (1/10) and 14% (1/7) in the control group. Medication scores (MS) showed a minimum 50% reduction in 20% of cats in both groups (3/15 in the test group and 2/10 in the control group) by 2 months. At 3 months, a 50% MS reduction was observed in 57% (8/14) of the test cats, but remained at 20% (2/10) for the control cats. Finally, at 6 months, 73% (8/11) of the test group cats had a minimum of 50% MS reduction, while in the control group, this figure was 17% (1/6).

Finally, when considering the number of cats in each group that achieved remission (remission defined here as a FeDESI score < 11), this was seen in 80% (12/15) of the test cats by 3 months and remained at 80% (8/10) when reaching 6 months. In the control group, 40% (4/10) of the cats achieved remission by 3 months and 50% (3/6) at 6 months.

## 4. Discussion

Feline Atopic Skin Syndrome is a common condition for which both diagnosis and effective management are challenging; its presentation is complex, and the condition is often lifelong and recurring in nature [28]. Drugs and other medicated approaches are available and typically form the primary source of treatment. However, these can bring cost and convenience issues, often referred to as caregiver burden [29], meaning owner compliance is variable. Allergen immunotherapy can also be effective in some cases and offers longer-term relief, notwithstanding the fact that identifying the allergen and optimising the protocol can be complex [30]. Therefore, strategies to reduce dependence on medications are likely to be welcomed by clinicians and pet owners.

Here, we have shown that a novel nutritional formula, in the form of a complete and balanced dry main meal, could benefit the clinical status and medication requirements of cats with FASS. We chose to focus our efficacy assessment on two established indicators of disease symptom severity and, as a surrogate measure, the level of medication judged necessary by the veterinarian for adequate symptomatic control. We have provided evidence that, over a six-month feeding period, a supplemented therapeutic diet was able to make a significant contribution to treatment efficacy when combined with conventional approaches. In the test diet group, symptom improvement, illustrated by FeDESI and VAScat, was observed relative to baseline measures at both 3 and 6 months. No such improvements were observed in the placebo diet group. Signs of benefit were further demonstrated by the differences observed between the diet groups at 3 months for FeDESI and at 3 and 6 months for VAScat. The lack of significant difference at 6 months for FeDESI (*p* = 0.2) merits further exploration, given that improvement was observed at the earlier timepoint. However, the more surprising aspect was that the above improvements in the test diet group were achieved against a backdrop of reduced drug administration. The average medication score decreased incrementally throughout the 6-month feeding period in this group and was, on average, less than 50% of the starting value by the end of the study. No significant reduction in medication was observed in the control diet group. It should be noted that the cases recruited for the study had mild and moderate disease severity, as judged by the starting FeDESI. These less severe cases may be the most amenable to dietary contributions. However, the results of this study provide evidence that the combination of nutritional components in the test diet formulation may modulate elements of the pathological mechanism responsible for causing and/or perpetuating feline atopic skin syndrome. This is one of the first practical demonstrations of such a role in the nutrition of cats. FASS is a complex condition whose presentation varies, and its biological background is still poorly characterised [9].

Current understanding suggests that there are at least some common features between FASS and atopic dermatitis in both dogs and humans [1]. Multiple factors likely contribute, including immune dysfunction [31,32,33] and host-microbiome interactions [34,35]. However, the role of the skin barrier in cats has not been extensively explored. The aim of a nutritional approach is to target multiple aetiological aspects of a disease simultaneously. The potential mechanisms of action of several components of the test diet formulation have been explored and reviewed elsewhere [16]. In brief, evidence supports the anti-inflammatory and antioxidant modes of action of turmeric. Bio-absorption of curcuminoids from turmeric is known to be limited; therefore, in the test diet, we employed a form of turmeric optimised for gut absorption [36], with the aim of increasing the delivery of bioactive compounds to target tissues. There is also evidence to support the anti-inflammatory role of omega-3 fatty acids, such as EPA and DHA, in atopic skin diseases [37]. Algal oil included in the tested diet was a rich source of these polyunsaturated fatty acids. It was selected owing to its greater sustainability compared to other sources of omega-3 PUFAs. Other active constituents, such as vitamin E, alpha-linoleic acid, and taurine, have shown promise in studies investigating dermatitis and skin barrier dysfunction [38,39,40]. Previous studies of this sort in other species have shown that the benefits of nutrition as part of the management of chronic complex conditions take time to manifest [18,19]. Typically, weeks and sometimes months are needed before improvements can be detected. In this instance, we were not able to collect a reliable follow-up data set earlier than 2 months for the medication score and 3 months for VAScat and FeDESI, the latter two occurring at the veterinary clinic. By this time, we were able to see the impact of the diet in the test group, in line with previous studies.

Although the results of this work are encouraging, there are acknowledged weaknesses in the design and execution of the study. Initial estimates based on data available from a similar canine study suggested that two groups of 20 would be required to provide an 80% chance of detecting a difference in the medication score at a 5% significance level. Recruitment of cats proved challenging, partly due to owners’ reluctance to commit to 6 months of monadic feeding, but also because the study was conducted during periods of COVID-related disruption. In addition, no attempt was made to balance the four FASS presentations at recruitment, indicating a random bias towards SIAH and HNP in the study population. Only one case of MD was included. SIAH, HNP, and EGC cats were represented in both diet groups, although SIAH was somewhat underrepresented in the control group. Owing to the low number of cats, it was not possible to explore the relative efficacy of the test and control diets for different presentations. The decision to assess the available data upon the completion of 25 cats was arbitrary and not based on any insights into the data at that time. In addition, vets and owners were not able to reliably compile data at monthly intervals; therefore, a decision was made to prioritise the collection of MS data monthly, whereas VAScat and FeDESI were collected at in-clinic appointments alone. It is also acknowledged that the medication score metric used for this study was originally intended for dogs and not cats [13]. As a consequence, the system was adapted from the canine model to better fit the needs of feline patients, acknowledging the differences in metabolism and drug efficacy between the two species. For example, the scores for Apoquel treatment reflect clinical observations and are intended to provide a consistent framework for evaluating therapeutic responses in cats. Quality of life questionnaires were originally circulated to owners as part of the project documentation, but few were completed, and those that were collected did not represent a usable dataset. Despite these suboptimal aspects, the results presented suggest a beneficial impact of a diet supplemented with a combination of fatty acids, antioxidants, and phytochemical extracts on cats with Feline atopic skin syndrome when fed for a minimum of 3 months.

## 5. Conclusions

Over a six-month feeding period, a novel, tailored cat diet was able to contribute to the clinical management of feline atopic skin syndrome. When compared to a placebo diet, the test food significantly reduced the drug requirement of FASS cats over the 6-month feeding period. In addition, the test diet reduced the symptomatic severity of skin lesions.

## Figures and Tables

**Table 1:** Medication Score determination scheme adapted from the scheme previously described by Fischer et al. [13]. NB. Apoquel was permitted as a medication at the veterinarians’ discretion, acknowledging its off-label status for use in cats at the time of writing. * Cefalosporin 20–30 mg/kg bid for 2–3 weeks; Amoxicillin and Clavulanic minimum 12.5 mg/kg bid.

Medication/Treatment	Dose/Schedule	Daily Score
No concurrent medication		0
Prednisolone/Dexamethasone	≥1 mg/kg/d	40
	0.5–1 mg/kg/d	30
	0.2–0.5 mg/kg/d	20
	≤0.2 mg/kg/d	10
Atopica (7 mg/kg bwt)	daily	30
	every other day	20
	every third day	10
	every fourth day	5
Apoquel (1 mg/kg)	twice daily	40
	daily	30
	every other day	20
	every third day	10
Antihistamine		10
Antibiotics (>21 days) *		20
Antibiotics (<21 days) *		10
Topical steroids		5
Topical hydrocortisone		5
Shampoo		5
Ear wash		5
Ear drops		5

**Table 2:** Key nutritional information for the two diets fed during the study. Dietary details are provided for macronutrients as well as other key ingredients that differed significantly between the test and control diets.

Diet Component	Units	Test	Control
Dry Matter	%	94.8	94.7
Moisture	%	5.2	5.3
Protein	%	33.5	33.2
Fat	%	22	22.5
Ash	%	6.7	6.6
Total Fibre	%	9.7	10.1
Linoleic Acid	%	4.4	2.9
EPA/DHA	%	0.55	0.034
Vitamin E	mg/kg	1120	536
Vitamin C	mg/kg	396	338
Taurine	mg/kg	4500	2600
Lutein	mg/kg	13	9
Curcuma	mg/kg	1425	0

**Table 3:** Distribution of FASS clinical presentations across the test and control diet groups.

Clinical Presentation	Test (n)	Control (n)
EGC	3	2
HNP	6	4
SIAH	2	0
MD	0	0
EGC/SIAH	2	3
HNP/SIAH	2	0
SIAH/MD	0	1

**Table 4:** Pairwise comparisons of outcomes between time points in each diet group. *p*-values are for baseline values minus values at Month 3 or 6. FeDESI, Feline Allergic Dermatitis Extent and Severity Index; VAScat, pruritic Visual Analogue Scale for cat; emmean, estimated marginal mean; effect sizes derived from the eff_size function from the emmeans package and are Cohen’s d effect sizes; CI, confidence intervals derived from the emmeans package.

Outcome		Emmean (95% CI); *p*-Values and Effect Sizes (95%) for Differences from Baseline					
		Test			Control		
		Baseline	3 mo	6 mo	Baseline	3 mo	6 mo
FeDESI	Emmean (95%CI)	17.35 (9.95–24.76)	7.92 (0.32–15.52)	4.94 (−4.04–13.91)	28.12 (18.32–37.92)	23.88 (14.07–33.69)	14.89 (2.41–27.34)
	*p*-value		0.037	0.02		0.64	0.09
	Effect size (95%CI)		1.01 (0.19–1.83)	1.33 (0.34–2.32)		0.46 (0.57–1.48)	1.42 (0.06–2.78)
VAScat	Emmean (95%CI)	7.2 (6.21–8.19)	4.35 (3.33–5.37)	3.81 (2.61–5.01)	5.89 (4.61–7.16)	5.56 (4.28–6.82)	5.35 (3.81–6.9)
	*p*-value		1.5 × 10^−4^	5.5 × 10^−5^		0.9	0.82
	Effect size 95%CI)		1.71 (0.88–2.53)	2.03 (1.1–2.97)		0.2 (−0.76–1.16)	0.32 (−0.77–1.41)

**Table 5:** Pairwise comparisons of the medication score outcome between time points in each diet group. *p*-values are for baseline values minus values at months 2, 3, 4, 5, or 6. emmean, estimated marginal mean; effect sizes derived from the eff_size function from the emmeans package, and are Cohen’s d effect sizes; CI, confidence intervals derived from the emmeans package.

Outcome		Emmean (95%CI); *p*-Values and Effect Sizes (95%) for Differences from Baseline					
		Test Group					
		Baseline	2mo	3mo	4mo	5mo	6mo
Medication score	Emmean (95%CI)	29.06 (21.53–36.6)	19.37 (11.85–26.9)	15.44 (7.81–23.07)	11.77 (4.03–19.49)	10.95 (3.11–18.81)	11.86 (3.74–19.99)
	*p*-value		0.02	3 × 10^−4^	3.8 × 10^−6^	2.2 × 10^−6^	2.5 × 10^−5^
	Effect size (95%CI)		1.15 (0.43–1.86)	1.61 (0.86–2.36)	2.04 (1.27–2.82)	2.14 (1.34–2.94)	2.03 (1.2–2.87)
		Control group					
		Baseline	2mo	3mo	4mo	5mo	6mo
	Emmean (95%CI)	28.00 (18.48–37.52)	23.00 (13.48–32.52)	24.5 (14.98–34.02)	22.51 (12.58–32.45))	19.33 (9.1–29.57)	32.78 (22.18–43.38)
	*p*-value		0.77	0.94	0.76	0.33	0.89
	Effect size (95%CI)		0.59 (−0.3–1.48)	0.41 (−0.47–1.3)	0.65 (−0.31–1.61)	1.02 (0.02–2.03)	0.57 (−1.62–0.49)

**Table 6:** Percentage of cats showing a minimum of 50% improvement in lesion severity (FeDESI), pruritus intensity (VAScat), and Medication Score after defined intervals of feeding the Test or Control diet.

Diet	Test					Control				
*Timepoint (mo)*	*2*	*3*	*4*	*5*	*6*	*2*	*3*	*4*	*5*	*6*
FeDESI		47%			50%		30%			67%
VAScat		40%			45%		10%			14%
Medication score	20%	53%	57%	69%	73%	20%	20%	50%	29%	17%

## Data Availability

Study data can be made available upon reasonable request to the authors.
